# Adiponectin mRNA Conjugated with Lipid Nanoparticles Specifically Targets the Pathogenesis of Type 2 Diabetes

**DOI:** 10.14336/AD.2024.0162

**Published:** 2024-05-15

**Authors:** Rady E. El-Araby, Qisheng Tu, Ying Xie, Tarek Aboushousha, Zhongyu Li, Xiaoyang Xu, Zoe X. Zhu, Lily Q. Dong, Jake Chen

**Affiliations:** ^1^Division of Oral Biology, Tufts University School of Dental Medicine, Boston, MA 02111, USA.; ^2^Theodor Bilharz Research Institute, Ministry of scientific Research, Cairo, Egypt.; ^3^Department of Chemical and Materials Engineering, New Jersey Institute of Technology, Newark, NJ, USA.; ^4^Department of Cell Systems and Anatomy, The University of Texas Health San Antonio, San Antonio, Texas 78229, USA.; ^5^Department of Genetics, Molecular and Cellular Biology, Tufts University School of Medicine, and Graduate School of Biomedical Sciences. 136 Harrison Ave, M&V 811, Boston, MA 02111, USA

**Keywords:** Type 2 diabetes, insulin resistance, Adiponectin, mRNA, Lipid Nanoparticle, Enhanced Endogenous APN

## Abstract

Type 2 diabetes (T2D) is a widespread health condition both in the United States and around the world, with insulin resistance playing a critical role in its development. Effective treatment strategies are essential for managing T2D and mitigating associated risks. Adiponectin (APN), secreted by adipocytes, exhibits an inverse correlation with obesity-related adiposity, and its levels are negatively associated with insulin resistance and body mass index. This study aimed to enhance endogenous APN levels in a diet-induced obese (DIO) mouse model using lipid nanoparticles (LNP) as safe delivery agents for APN mRNA conjugates. The results indicate that APN-mRNA-LNP administration successfully induced APN synthesis in various tissues, including muscle, liver, kidney, pancreas, and adipose cells. This induction was associated with several positive outcomes, such as preventing diet-induced body weight gain, improving hyperglycemia by promoting Glut-4 expression, alleviating diabetic nephropathy symptoms by blocking the EGFR pathway, and reducing pro-inflammatory cytokine production. In addition, the treatment demonstrated enhanced insulin sensitivity by activating DGKd and inhibiting PKCε. This resulted in reactivation of insulin receptors in insulin target tissues and stimulation of insulin secretion from pancreatic beta cells. The findings of the present study highlight the potential of APN-mRNA-LNP-based nucleic acid therapy as a treatment for type 2 diabetes, offering a comprehensive approach to addressing its complexities.

## INTRODUCTION

The prevalence of diabetes in the United States has reached alarming proportions. About 34.2 million Americans, constituting 10.5% of the population, currently suffer from diabetes, according to the Centers for Disease Control and Prevention's (CDC) 2020 National Diabetes Statistics Report https://diabetesresearch.org. Moreover, in 2018 alone, over 1.5 million new cases of diabetes were diagnosed in individuals aged 18 years and older in the United States, encompassing more than 210,000 children and teenagers under the age of 20 [1]. Globally, an estimated 537 million individuals are affected by diabetes, with most cases attributed to type 2 diabetes (T2D) [1, 2]. Key elements in the pathogenesis of T2D include insulin resistance and early hyperinsulinemia, which contribute to a gradual decline in the ability of pancreatic β-cells to produce insulin. The intricate interplay between β-cell dysfunction and insulin resistance underscores the complexity of T2D [3]. Consequently, T2D emerges as one of the most pervasive metabolic diseases on a global scale.

Adipocytes are no longer viewed as passive fat deposits, but rather as dynamic, multifunctional tissues that play a role in maintaining energy substrate balance and complex secretory processes related to nutritional status [4, 5]. Over time, these cells have evolved from passive repositories of fat into active endocrine organs [6]. Adipokines, bioactive chemicals produced by adipocytes, play an important role in this endocrine function [7]. Many adipocytokines are negatively impacted by the altered endocrine activities of adipose tissue, with adiponectin (APN) being one of the most important proteins secreted by adipocytes [8].

APN, also known as Acrp30, AdipoQ, ApM1, and GBP28 [9], is a 30 kDa monomeric protein encoded by the ADIPOQ gene on chromosome 3q27, spanning approximately 15.8 kb. This protein is associated with a susceptibility locus for cardiovascular disease, type 2 diabetes, and metabolic syndrome [10]. Similar to leptin [11], monomeric APN is primarily produced and released in white adipose tissue. Glycosylation and hydroxylation play a critical role in controlling its activity and receptor binding [12].

In the context of obesity, adiponectin levels have an inverse relationship with adipose tissue. In normal-weight individuals, adiponectin levels are typically between 2 and 30 ng/ml [13]. As seen in individuals with balanced body composition, adipocytes, and immune cells, APN levels are negatively correlated with insulin resistance and BMI [8, 14]. Obesity-related adipose tissue growth triggers an inflammatory profile that lowers adiponectin secretion and levels [7]. Chronic inflammatory diseases such as T2D, obesity, and atherosclerosis are associated with decreased serum APN concentrations [8, 15, 16]. There is also evidence that adiponectin deficiency plays a role in T2D pathogenesis [17, 18].

mRNA has shown great therapeutic potential in a wide range of applications, including viral vaccines, protein replacement therapies, cancer immunotherapies, cellular reprogramming, and genome editing. Its versatility makes it a promising tool for a variety of therapeutic approaches [19-30]. Efficient mRNA delivery has been the focus of extensive research, which has led to the development of various materials, including lipids, lipid-like compounds, polymers, and protein derivatives [20, 21, 23-30]. Among these, lipid nanoparticles (LNPs) have attracted considerable attention due to their efficacy in transporting small molecules [31], siRNA drugs [31], and mRNA [32-34]. LNPs have shown promising results and found successful applications in clinical settings, such as the mRNA-1273 [32, 34] and BNT162b21 vaccines for coronavirus disease 2019 (COVID-19) [35].

There are numerous interacting pathways involved in type 2 Diabetes (T2D), and the present study focuses on these pathways. The activation of adiponectin receptors (AdipoR1/AdipoR2) and subsequent association with adapter proteins facilitate glucose uptake through APPL1-Rab5 or APPL1-AMP-AMPK-mediated translocation of glucose transporter 4 (GLUT4), resulting in the suppression of insulin signaling [36]. Subsequent impairment of adiponectin signaling mechanisms, particularly GLUT4 translocation, impedes cellular glucose uptake for energy production [37]. Insulin resistance is commonly characterized by decreased GLUT4-dependent glucose absorption in skeletal muscles and adipose tissue [38].

A temporal increase in intracellular diacylglycerol (DAG) mass is associated with glucose-induced insulin resistance [39]. Prolonged elevation of intracellular DAG activates protein kinase C (PKC) isoforms, leading to insulin resistance, intracellular lipid accumulation, and impaired signal transmission [40, 41]. Glucose transport is downregulated due to increased PKC-mediated serine phosphorylation of the insulin receptor (IR) and insulin receptor substrate 1 (IRS-1) [42, 43]. Chibalin et al. [43], in their study, suggest that controlling hyperglycemia can reverse the decline in diacylglycerol kinase delta (DGKd) protein and DGK kinase activity. This could provide pharmacological strategies that target DAG and PA metabolism through DGKd modulation, potentially preventing and controlling insulin resistance in metabolic disorders [43].

Moreover, insulin resistance and diabetic nephropathy (DN) in T2D are associated with epidermal growth factor receptor (EGFR) activation. This activation increases immune cell infiltration and oxidative stress in the kidney and adipose mass while simultaneously reducing pancreatic insulin synthesis and adipocyte adiponectin production [44]. Inhibition of EGFR decreases the expression of proinflammatory cytokines (iNOS, TNF-α, INF-γ, IL-6) [45, 46]. Based on these insights, the present study aims to explore the possibility of replicating these steps *in situ*. The present study aims to assess the efficiency of APN-mRNA-LNP (a delivery system comprising adiponectin mRNA conjugated with lipid nanoparticles (LNP)), in stimulating endogenous production, evaluating APN expression *in situ*, and examine downstream signaling cascades associated with T2D.

## MATERIAL AND METHODS

### Cell culture

C2C12: Mouse C2C12 muscle cells were obtained from the American Type Culture Collection (ATCC CRL-1772) (Manassas, VA). The myoblasts were seeded in Dulbecco’s modified Eagle medium (DMEM, No. 2492921, Gibco) as growth medium (GM), which contained D-glucose (HG, 4.5 g/l) and L-Glutamine, supplemented with 10% fetal bovine serum (FBS) and 1% (100 U/ml) penicillin/ streptomycin (P/S) under humidified atmospheric conditions at 37°C and 5% CO_2_. Subculturing was done by trypsinization, and cells were diluted to 2 x 10^5^ cells/ml and seeded in plates. To induce C2C12 differentiation (myotubes) a day after the cells reached 70% confluence, the medium was changed to differentiation medium (DM) (DMEM with 2% horse serum and 1% P/S) and maintained in DM thereafter. The differentiation media was changed every 48 hours, and up until the fifth day of differentiation induction, the establishment of polynucleotide myotubes was evaluated microscopically [47].

SV40 MES13: Mouse mesangial glomerulus SV40-MES13 kidney cells were obtained from the American Type Culture Collection (ATCC CRL-1927) (Manassas, VA). SV40 MES13 were cultured in 3:1 mixture of ATCC-formulated Dulbecco’s Modified Eagle’s medium (DMEM, No. 30-2002, ATCC) and F12 contained GlutaMAX-1 Ham (Nutrition Mixture, No. 2193045, GIBCO), supplemented with 5% FBS and 1% (100 U/ml) P/S under humidified atmospheric conditions at 37°C and 5% CO_2_. The complete medium was replaced every 48 h and subcultured at a ratio of 1:5 [48]. All the cell experiments were performed below passage number 10 (C2C12) or 7 (SV40 MES13) in a humidified environment at 37°C and 5% CO_2_.

High glucose (HG) stress: The cells grew to subconfluency until they reached the required cell number for the experimental setup. 3.5 × 10^5^ differentiated C2C12 cells on day 6 and 2.0 × 10^6^ SV40 MES13 were seeded into 6- well plates after 24 hours, when cells had reached approximately 70% confluence. Thereafter, the cells were assigned into the two study groups, negative control group and positive group, which were cultured with high glucose 10 g/l (55 mM) (D-glucose, No. A24940-01, GIBCO, (www.sigmaaldrich.com/US/en/technical-documents) for 72 hours without medium changes [49].

### APN mRNA Modification and encapsulation in LNP

Both the formulation and the APN messenger RNA were subjected to chemical modification (cmRNA). Briefly, T7 RNA polymerase *in vitro* transcription produced cmRNA encoding APN (IVT). The full-length mRNA for APN (NM 009605) was produced (BioSynthesis, Inc., USA). The mRNA APN was chemically altered by ribonucleotide substitution, in which uridine residues were replaced with pseudouridine and cytidine residues with 5-methylcylidine to increase RNA stability while reducing the anti-RNA immune response. To ensure effective translation by the intended cells, a poly(A) tail (extending to 120 nucleotides) and a 7-methylguanylate cap (at the 3 and 5 ends, respectively) were also added. The quantity and quality of cmRNA APN was assessed using the NanoDrop 2000C (Thermo Fisher Scientific, USA). The purity and size were confirmed by automated capillary electrophoresis using a Fragment Analyzer (Advanced Analytical, USA). High-performance liquid chromatography was used to analyze the nucleotides (BioSynthesis, Inc., USA). To generate APN mRNA contained in LNPs given by the Co-I Dr. Xu's lab, a reliable self-assembly approach was used in our experimentation [50, 51]. Briefly, AA3-DLin LNPs with a molar ratio of 40:40:25:0.5 (AA3-DLin: DOPE: Cholesterol: DMG-PEG) were used to deliver APN mRNA for the following studies. The preparation and characterization of AA3-DLin LNPs used in this study were well-established and evaluated in previous publications [51-53]. In addition, the mCherry encoded mRNA purchased from Trilink was encapsulated into AA3-DLin LNPs to transfect Hek 293 cells. The mCherry positive cells were then observed under a fluorescent microscope for intracellular uptake investigation. The physicochemical properties, transfection evaluations, and intracellular uptake of the nanoparticles are shown in Supplemental Figure 2.

### APN-mRNA-LNP Cytotoxicity

Cytotoxicity of APN-mRNA-LNP was assessed by cell viability assay in C2C12 differentiated myotubes. C2C12 cells were harvested after 6 days of differentiation and seeded in 96-well plates at a density of 7.0 × 10^4^. The myotube cells were treated with APN-mRNA-LNP at various concentrations (0-64) µg/ml for 24 h in blank medium, in parallel with blank wells, and in an Empty-LNP well. Cell viability assays were determined using the manufacturer’s protocol of Cell Counting Kit-8 (CCK8) (No. TS511, Dojindo, Japan). Briefly, 10 ml of WST-8 were added to each treated cell and incubated for 3 h at 37°C. Thereafter, absorbance was measured at 450 nm. The half maximal inhibitory concentration (IC50) for APN-mRNA-LNP was calculated depending on the readings of the CCK-8 test using the AAT Bioquest online tools (www.aatbio.com).

### APN-mRNA-LNP administration

*In vitro:* For APN-mRNA-LNP transfection, cells were cultured in high-glucose media “HG stress 10 g/L (55mM D-glucose)” for 72 h, then the media were replaced with blank media. One µg/ml APN-mRNA-LNP was added to three treated plates (24, 48, and 72 h), as well as to parallel plates containing PBS as a control, a negative control (seeded in normal media) and a positive control (PC) (seeded in HG media), and an Empty-LNP group (seeded in HG media). Cells were extracted after 24 h of transfection for gene and protein expression analysis.

Experimental Animals: All *in vivo* procedures were approved by the Institutional Animal Care and Use Committee (IACUC) at Tufts University. DIO (C57BL/6J) male mice (Strain #:380050, RRID: IMSR-JAX: 380050), were purchased from The Jackson Laboratory (600 Main Street Bar Harbor, ME USA 04609) at 6 weeks of age. Starting at 6 weeks-old, the mice were fed a high-fat diet (HFD) (D12492, Research Diets, NJ. USA) containing 60 kcal/g of fat for approximately 19-21 weeks. Mice were maintained at constant temperature (23 ± 2 °C), humidity (45-55%), and a 12-h light/dark cycle with *ad libitum* access to water and food (www.jax.org/strain/380050). Blood glucose and body weight were assessed weekly to ensure that the rats had acclimated for at least 2 weeks prior to the start of the study.

### Glucose and insulin tolerance tests

At 23 weeks, the mice were assigned randomly into the different study groups (n=5) based on random selection via "lottery design" in an unbiased, "without prejudice"manner. The intraperitoneal glucose tolerance test (IPGTT) and insulin tolerance test (ITT) were carried out to assess insulin sensitivity and glucose tolerance, respectively, in order to prevent significant variations between groups.

After 12 hours of fasting, the IPGTT was used to evaluate glucose tolerance (21:00-9:00). Blood samples were obtained from the mice's tails at 0, 30, 60, 90, and 120 min following an intraperitoneal injection of 50% D-Glucose solution 200 g/l (No. 2423423, GIBCO) at a dose of 2 g/kg. Thereafter, a blood glucose test was performed [54]. Insulin sensitivity was evaluated using ITT. Because insulin rapidly lowers blood glucose levels, mice were fasted for only 6 h (7:00-13:00) prior to ITT. The mice were weighed prior to receiving an intraperitoneal injection of 0.75 IU/kg body weight of isophane insulin suspension 100 U/ml (No. 0002-8315, Lilly). At 0, 30, 60, 90, and 120 min, blood was drawn from the tail and the blood glucose level was assessed [54]. A Clarity Plus Blood Glucose Meter was used to measure the blood sugar (Clarity Diagnosis; China).

### APN-mRNA-LNP Injection

Male C57BL/6J mice on a high-fat diet (HFD) received 0.3 mg/kg (~10 ug/mouse) of APN-mRNA-LNP intravenously once at 25 weeks of age. The control group received the same dose of phosphate buffered saline (PBS), while the second positive LNP group received Empty-LNP at the same time. To avoid any noticeable variations in blood glucose levels or body weight prior to the evaluation, measurements taken before the baseline day (APN-mRNA-LNP injection) were compared to measurements taken 2 weeks, 1 week, or 3 days prior to and after injection.

After 3 days, 1 week, and 2 weeks of APN-mRNA-LNP injection, mice were anesthetized with ketamine/xylazine 10 mg/kg intraperitoneally. For the target engagement study, the liver, skeletal muscles, kidney, pancreas, W. fat, and B. fat tissues were collected and directly placed in liquid nitrogen before being stored at -80°C for mRNA analysis. The kidney, pancreas, liver, and skeletal muscles were weighed for the efficacy stduy and thereafter preserved in formalin according to the protocol outlined below for HE staining.

### Gene expression evaluation

After transfection in the cell lines studied, total RNA was extracted using Quick-RNA Miniprep Kit (ZYMO Research, Irvine, CA, USA). However, Trizol reagent (Ambion, Life technology, CA, USA) was used to extract the total RNA from tissue samples. One μg of total RNA was subjected to reverse transcription using the M-MLV Reverse Transcriptase (Thermo Scientific, Waltham, MA, USA) according to the manufacturer’s protocol. Using primers of the investigated genes, PowerUp SYBR Green Master Mix (Thermo Scientific) was utilized for real-time quantitative PCR (qRT-PCR), which was carried out on a Bio-Rad iQ5 thermal cycler (Bio-Rad Laboratories, Hercules, CA, USA) (Supplemental Table 1). Differences in expression were evaluated by the comparative cycle threshold method using Glyceraldehyde 3-phosphate dehydrogenase (GAPDH) as a control.

### Western Blot

After transfection, the cells were collected directly in 100 μl protein sample buffer (#2463560, Invitrogen) and then denatured at 90°C for 10 min. Individual samples of 15 μl were loaded in each lane. Thereafter, western blotting was performed according to standard protocols. The primary antibodies used were APN (#2789, Cell signaling), Glut-4 (#PA5-80022, Invitrogen), DGK-d (#PA5-75256, Invitrogen), PKCε (#PA5-32715, Invitrogen), TNF-α (#11948, Cell signaling) and IL-6 (#12912, Cell signaling). An anti-rabbit IgG (HRP-linked antibody) was used as a secondary antibody (#7074S, Cell Signaling). Three separate blot analysis of the samples were conducted. Optical densitometry was used to semiquantify the bands, and ImageJ digital imaging processing software was used for analysis (ImageJ 1.53t, National Institutes of Health, Bethesda, MD, USA). GAPDH was used as an endogenous control to normalize the expression of each protein under investigation.

### Histopathological studies

The target tissues (liver, kidney, skeletal muscles, and pancreas) were obtained from the experimental animals after sacrifice and fixed in a 10% buffered formaldehyde solution for 24 h to perform histological examinations. The tissues were sectioned using a rotary microtome following graded alcohol dehydration, cleaning, and embedding.

Hematoxylin and Eosin (H&E) were used to stain the acquired sections, and an Olympus DP73 microscope with objective powers of 20, 40, and 100 were used to analyze the results.

Periodic Acid Schiff (PAS) Stain: The kidney was fixed in paraffin and 3-μM-thick sections were prepared for PAS staining. PAS was performed using PAS-IFU Stain Kit Modified Lillie’s (ScyTek Laboratories, INC., West Logan, UT, USA) following the manufacturer’s recommendations.

### Immunohistochemistry (IHC)

IHC was generated from 5-M-thick sections of paraffin-embedded liver, skeletal muscles, and kidney tissues. The Histostain-SP Kit (Life Technologies, Waltham, MA, USA) was used according to the manufacturer's instructions for IHC. It includes the LABSA method, which uses an affinity-purified and biotinylated secondary antibody that binds specifically to the primary antibody. Protein Tech supplied the adiponectin primary antibody (1:400; #21613-1-AP, Rosemont, IL, USA). Stained tissues were captured digitally with an Olympus DP73 microscope.

### Statistical analysis

A statistical package for social sciences (SPSS) version 29.0.1.1 (244) of IBM SPSS statistics was used for the analysis of the data. Data were presented as mean SD with a 95% confidence interval; a p value 0.05 was considered statistically significant. In order to determine the normality of the data, the Shapiro-Wilk normality test was performed (n > 6). In vitro samples were biologically and technically triplicated (n=3x3=9). Based on power analysis, the number of animals required per group was derived, and although technically triplicated, the total number of in vivo samples was (n = 3x5=15). To compare the means of normally distributed variables between groups, Student's (t) tests were used. For non-parameteric variables, Mann-Whitney U tests were used (n<6). Using the Pearson correlation coefficient (r), a correlation coefficient was calculated. The area under the curve (AUC) was calculated as an accuracy metric in order to evaluate the IPGTT and ITT sensitivity of selected tests calculated using the trapezoid method. Western blots were done to evaluate the protein expression in the samples. Three separate blot analyses were performed on each sample. A semi-quantitative analysis of the bands was conducted using optical densitometry and ImageJ (1.53t) software for digital imaging processing. Protein expression has been controlled by GAPDH as an endogenous control. Levels of protein are expressed as mean ratios between protein bands of targeted protein and GAPDH in comparison with a negative control.

## RESULTS

### The cytotoxicity of APN-mRNA-LNPin In-vivo study

**Figure 1.**
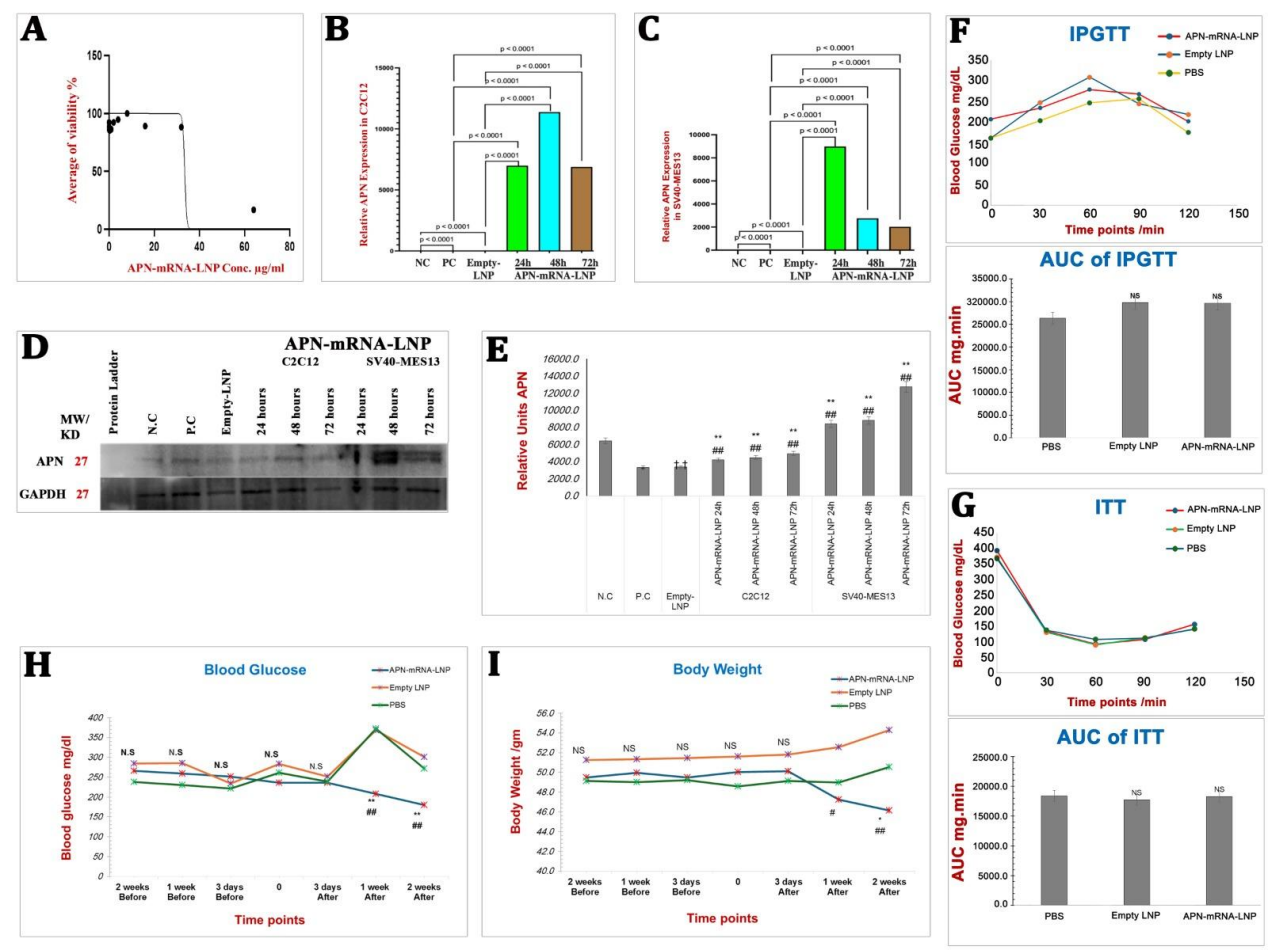
**APN evaluation *in vitro*, with *in vivo* measurement of IPGTT, ITT, blood glucose and body weight *in vivo.* (A) C2C12 myotubes viability was assessed using the CCK8 assay**. After transfection with APN-mRNA-LNP, the half maximal inhibitory concentration (IC50) was determined after 24 hours. (**B**) Relative APN Expression in differentiated Myotubes cell C2C12, (C) Relative APN Expression in Kidney mesangial cell SV40-MES13. APN gene expression was calculated based on the Law of Fold-change, which is 2^-ΔΔCT^. Fold-change values less than one indicate downregulation, whereas values greater than one indicate upregulation. Both biological and technical triplicates were performed *in vitro*. Values are mean ± SD. n = 9, student t-test. (**D**) APN protein expression, three separate blot analyzes of the samples were conducted. (**E**) Optical densitometry was used to semi-quantify the bands, and ImageJ (1.53t) digital imaging processing software was used for analysis. GAPDH has been used as an endogenous control for protein expression. Protein levels are represented as mean ratio values quantified from protein bands of APN versus GAPDH compared to negative control. (n = 3, Mann-Whitney U tests), Significances are shown above bars: ^††^P < 0.01 vs. negative control (NC), ^**^P < 0.01 vs. positive control (PC) “PBS” and ^##^P < 0.01 vs. Empty-LNP. (**F**) IPGTT with AUC of IPGTT, (G) ITT curve with AUC of ITT. The IPGTT and insulin ITT were utilized at 23 weeks old, to evaluate glucose tolerance and insulin sensitivity, respectively to depict significant changes between groups, the area under the curve (AUC) of IPGTT and ITT was calculated by the trapezoid method. Values are mean ± SD. n = 5 each group, Mann-Whitney U tests, ^**^ P < 0.01 vs. PBS control group, NS; Non-significant. H) Blood glucose levels at different time points were evaluated by glucometer; I) Body weight at different time points. Values are mean ± SD. n = 5 each group, Mann-Whitney U tests, ^**^ P < 0.01 vs. PBS control group and ^##^P < 0.01 vs. Empty-LNP group.

The cytotoxicity of APN-mRNA-LNP on C2C12 myotubes was investigated at different concentrations (ranging from 0 to 64 µg/ml) over a period of 24 h. Notably, no cytotoxic effects were observed at a concentration of 32 µg/ml. The calculated IC50 value after a 24-h period was found to be 42.56 μg/mL (Fig. 1A).

### Increased APN expression in APN-mRNA-LNP treated C2C12 and SV40-MES13 cells

Expression of the APN gene significantly increased over time upon exposure to 1 μg/ml APN-mRNA-LNP in C2C12 myotubes (Fig. 1B) and SV40-MES13 kidney mesangial cells (Fig. 1C) after 24, 48, and 72 h of transfection, as compared with the PC and Empty-LNP groups. Western blot analysis was used to assess APN protein expression, and the results showed that the PC and Empty-LNP groups had considerably lower levels of APN protein expression than the NC group (p < 0.001). On the other hand, both C2C12 myotubes and SV40-MES13 kidney mesangial cells showed significantly higher APN protein expression after 24, 48, and 72 h of transfection as compared with the PC and Empty-LNP groups (p < 0.001) (Fig. 1D, E).

*APN-mRNA-LNP Administration Leads to Reduction in Blood Glucose Levels and Attenuated Body Weight Gain in DIO Mice*

Mice were fed with a high-fat diet (HFD) and tested at 23 weeks of age using the insulin tolerance test (ITT) and the intraperitoneal glucose tolerance test (IPGTT). These tests aimed to assess glucose tolerance and insulin sensitivity before the administration of APN-mRNA-LNP. Conducting these tests before injection ensured a comparable baseline among the groups. The area under the curve (AUC) for both IPGTT and ITT was calculated using the trapezoid method. No significant differences in glucose tolerance were observed, as the AUC of IPGTT did not differ significantly as compared with the PBS control mice (Fig. 1F). Similar results were also observed for ITT, with no significant differences in insulin resistance between groups indicated by the AUC of ITT (Fig. 1G).

**Figure 2.**
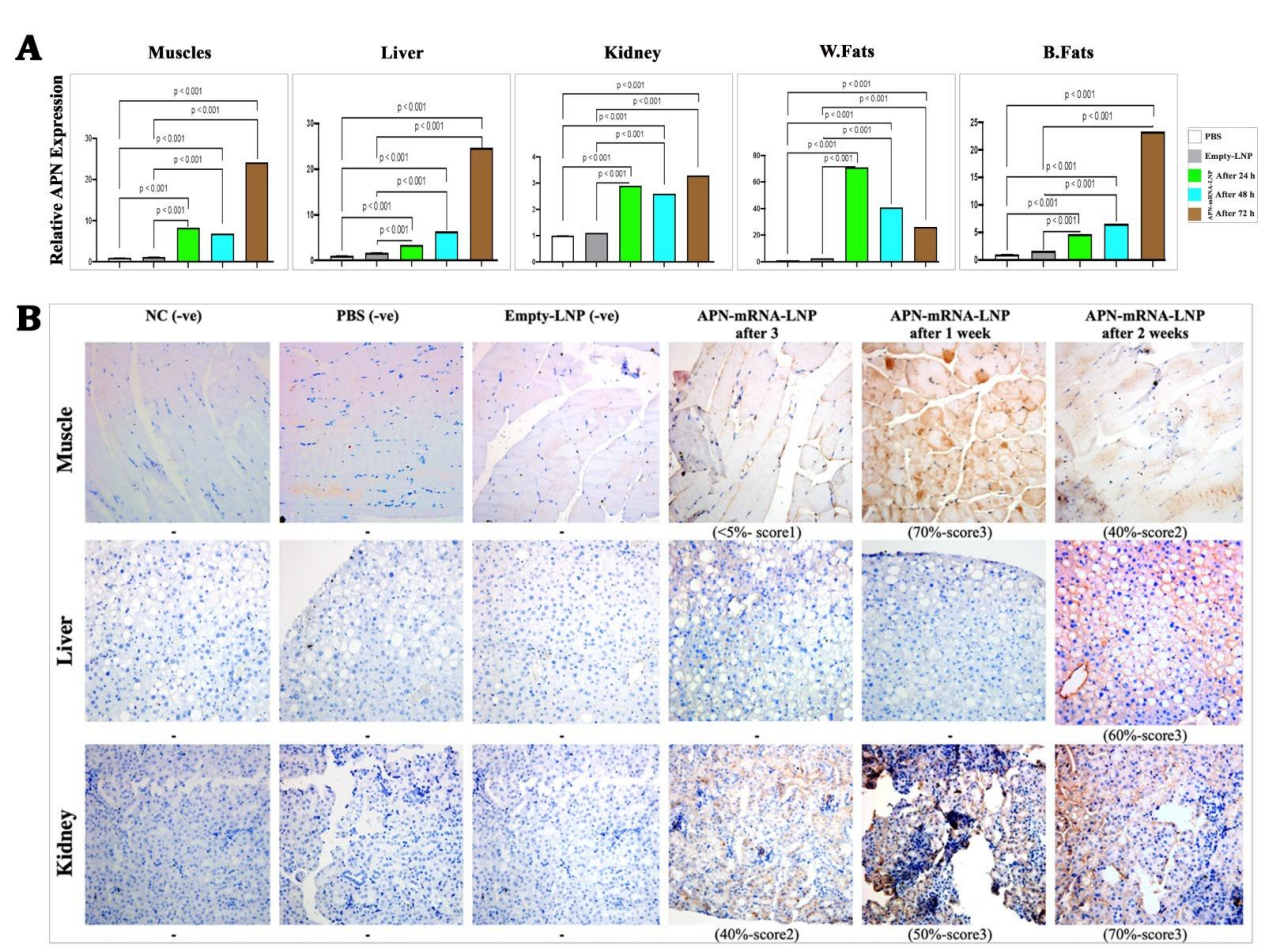
**APN *in situ* expression**. (**A**) APN gene expression in different tissues. Skeletal muscles; Liver; Kidney; White fats; Brown fats. APN gene expression was calculated based on the Law of Fold-change, which is 2^-ΔΔCT^. Fold-change values less than one indicate downregulation, whereas values greater than one indicate upregulation. *In vivo*, a total of five biological samples (n = 5) were collected. Of each sample, three replicates were performed technically., ^**^ P < 0.01 vs. PBS control group and ^##^P <0.01 vs. Empty-LNP group. (**B**) Immunohistochemical staining for adiponectin in T2D skeletal muscles, liver, and kidney mice in the studied groups. Negative controls were performed by blocking PBS without APN-Ab. Treated groups were performed by blocking with APN-Ab (200X). Labeling index was used to determine APN-Ab percentile expression (the ratio of positively stained cells/total cells × 100). Blinded staining intensity scores were assigned to each specimen based on arbitrary values of 1, 2, or 3 (reflecting weak, intermediate, and bright staining). A total of five biological samples (n = 5) were collected; three sections were taken from each sample. Scale bar= 50 μm.

At 25 weeks of age, male C57BL/6J mice on a high-fat diet (HFD) received intravenous administration of 0.3 mg/kg (~10 µg/mouse) APN-mRNA-LNP. No significant differences in blood glucose levels were observed between groups until the third day after injection. However, one week after the injection, a notable decline in blood glucose levels occurred. The blood glucose level reached 207.9±31.3 mg/dl, which was significantly lower than the PBS control group (372.3±69.0 mg/dl, p < 0.001) and the Empty-LNP group (368.3±28.9 mg/dl, p < 0.001). It is worthy of note that blood glucose levels further decreased to 180.0±18.2 mg/dl two weeks after injection, as compared with 271.3±38.0 mg/dl for the PBS control group and 301.0±42.6 mg/dl for the Empty-LNP group (Fig. 1H). In addition, body weight was measured during the two-week period before and after injection. No significant difference was observed between 2 weeks before injection and 3 days after injection. However, a decrease in body weight was seen one week after injection, reaching 47.2±2.5 gm; this difference was statistically significant (p = 0.014) when compared to Empty-LNP (52.6±3.8 gm). After two weeks, the body weight further decreased to 46.1±1.7 gm, with a p value of 0.05 when compared with the PBS control group (50.6±3.5 gm) and Empty-LNP group (54.3±1.7 gm) with a p value of 0.001 (Fig. 1I).

### The administration of APN-mRNA-LNP increased APN expression in situ

Regarding gene expression, the evaluation of APN expression in the studied groups was performed after three days, one week, and two weeks of injection. After three days of injections, APN expression significantly increased (p < 0.001) by 8.31-fold as compared with the PBS and Empty-LNP groups. This increase persisted after one week (6.93-fold) and further intensified after two weeks, reaching 24.18-fold changes in skeletal muscle tissue samples (Fig. 2A). A similar trend was observed in liver tissue, where APN increased by 3.38-fold after three days, 6.36-fold after one week, and 24.75-fold changes after two weeks (p < 0.001) as compared with the PBS and Empty-LNP groups (Fig. 2A). While APN expressions were slightly higher in kidney and pancreas samples, there were 2.88- and 2.55-fold changes after three days, respectively. In addition, there were 2.59- and 3.11-fold changes after one week, and 3.27- and 3.13-fold changes after two weeks (Fig. 2A), respectively, with a significance level of p < 0.01 when compared with the PBS and Empty-LNP groups. In adipose tissues, especially in white fat tissues (W. fats), APN showed remarkable overexpression after three days, reaching 71.21, 40.80 after one week and 26.11-fold changes after two weeks (p < 0.001) when compared with the PBS and Empty-LNP groups (Fig. 2A). In brown fats (B. fats), APN increased gradually after three days to 4.68, 6.53 after one week, and reached 23.33 after two weeks (p value < 0.001) when compared with the PBS and Empty-LNP groups (Fig. 2A).

In the untreated groups (PBS and Empty-LNP), the adiponectin protein was not expressed in muscle, liver, and kidney samples. However, following 3 days, one week, and two weeks of APN-mRNA-LNP administration, skeletal muscles showed a significant increase in adiponectin expression (5, 70, and 40%), along with comparable intensity scores (1, 3, and 2). In kidney tissue, adiponectin expression increased gradually (40, 50, and 70%) with intensity scores (2, 3, and 3), respectively. In liver tissue, adiponectin was expressed after two weeks of injection (60% with a score of 3 on the intensity scale). The administration of APN-mRNA-LNP resulted in *in situ* adiponectin expression in the muscles, kidney, and liver of DIO mice (Fig. 2B).

### APN-mRNA-LNP treatment affects T2D pathways following increased APN expression

The present study hypothesis focused on the analyzes of seven axes directly associated with Type 2 Diabetes (T2D), including Glut-4 reactivation, improvement in the Insulin Resistant Pathway (DGKd and PKCε), IR activation, activation of insulin secretion through the reactivation of the island of Langerhans EGFR Pathway Inhibition, Reduction of Inflammatory Cytokines (TNF-α, IL-Ib, IL-6), andReduction in Fatty Changes. The expected gene expression in various studied tissues is summarized in Supplemental Fig. 1, utilizing information from the Mouse Genome Database (MGD) and the Gene Expression Database (GXD).

We investigated the correlation between APN overexpression and targeted 7-axis pathways by evaluating mRNA and protein expression in SV40-MES13 and C2C12 cell lines. In addition, *in vivo* effects were observed 3 days, 1 week, and 2 weeks after APN-mRNA-LNP administration.

*Glut-4 reactivation (glucose uptake improvement)*

*In vitro*, the results showed a significant increase in Glut-4 gene expression after APN-mRNA-LNP transfection for 24, 48, and 72 h in C2C12 myotubes (p < 0.001 at all timepoints) and SV40-MES13 kidney mesangial cells (p < 0.001, 0.001, and 0.01, respectively), as compared with the PC and Empty-LNP groups.

**Figure 3.**
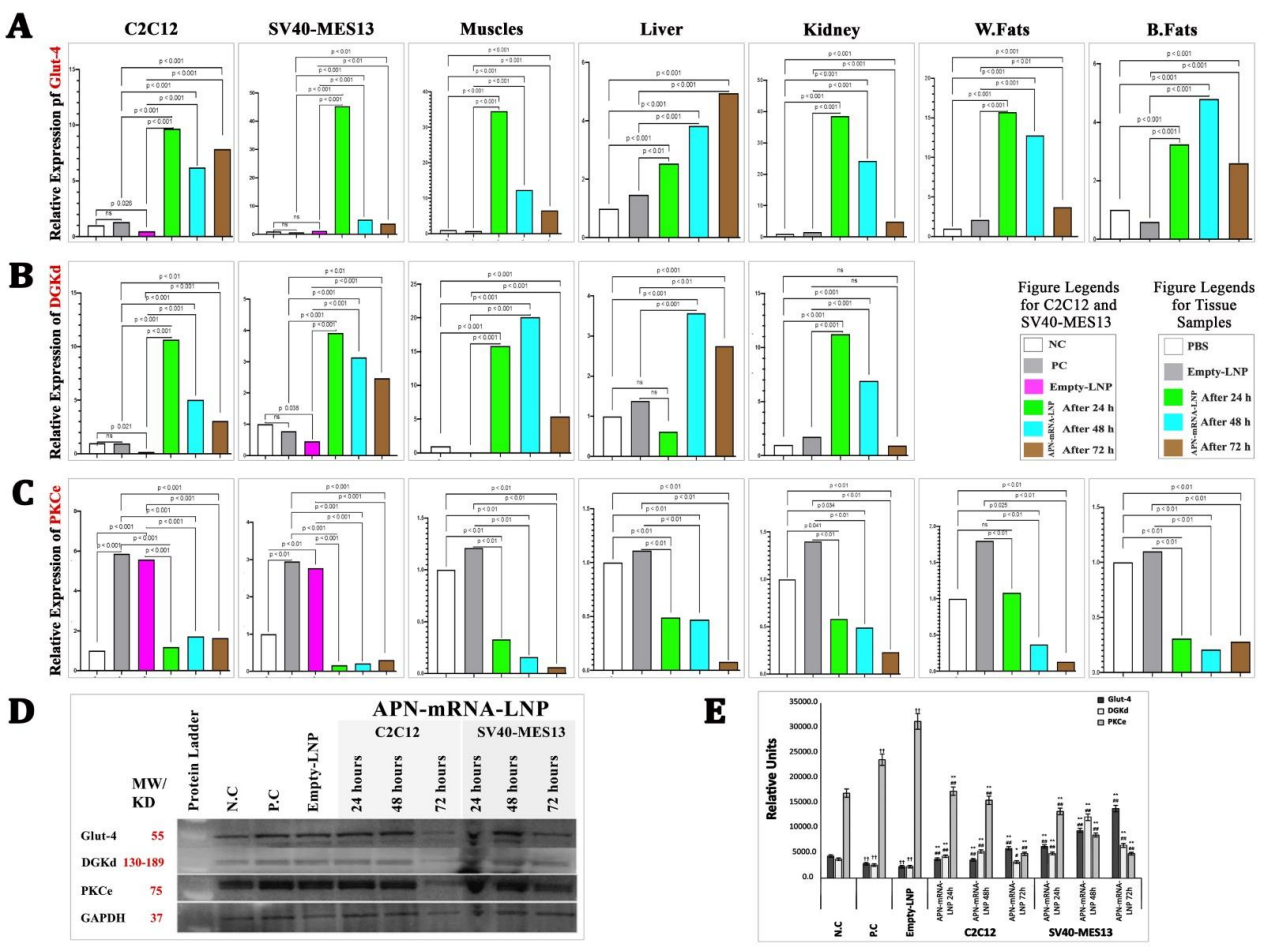
**Relative gene expression in different tissues**. (**A**) Glut-4 gene in differentiated myotubes cell C2C12, Kidney mesangial cell SV40-MES13, Skeletal muscles, Liver, Kidney, White fats, and Brown fats. (**B**) DGKd gene in differentiated myotubes cell C2C12, Kidney mesangial cell SV40-MES13, Skeletal muscles, Liver, and Kidney. (**C**) PKCe gene in differentiated myotubes cell C2C12, Kidney mesangial cell SV40-MES13, Skeletal muscles, Liver, Kidney, White fats, and Brown fats. Gene expression was calculated based on the Law of Fold-change, which is 2^-ΔΔCT^. Fold-change values less than one indicate downregulation, whereas values greater than one indicate upregulation. Both biological and technical triplicates were performed in vitro. Values are mean ± SD. n = 9, student t-test. In vivo, a total of five biological samples (n = 5) were collected. Of each sample, three replicates were performed technically. (**D**) Glut-4, DGKd, and PKCe protein expression, three separate blot analysis of the samples were conducted. (**E**) Optical densitometry was used to semi-quantify the bands, and ImageJ (1.53t) digital imaging processing software was used for analysis. GAPDH has been used as an endogenous control for protein expression. Protein levels are represented as mean ratio values quantified from protein bands of the targeted protein versus GAPDH compared to negative control. (n = 3, Mann-Whitney U tests), Significances are shown above bars: ^††^P < 0.01 vs. negative control (NC), ^**^P < 0.01 vs. positive control (PC) “PBS” and ^##^P < 0.01 vs. Empty-LNP.

*In vivo*, Glut-4 gene expression showed significant improvement in skeletal muscles, liver, kidney, W. fat, and B. fat tissues after 3 days, 1 week, and 2 weeks of APN-mRNA-LNP injections as compared with the PBS and Empty-LNP groups (Fig. 3A).

For Glut-4 protein expression, it was significantly lower in the PC and Empty-LNP groups as compared with the NC group (p < 0.001). However, when the treated groups were compared with the PC and Empty-LNP groups, Glut-4 protein expression was significantly higher after 24, 48, and 72 h of transfection in C2C12 myotubes (p < 0.001 at all timepoints) and in SV40-MES13 kidney mesangial cells, exhibiting a significant gradual increase (p < 0.001) at all timepoints (Fig. 3D, E). These findings indicate that both in the examined cell line and the diabetic mouse model, the injection of APN-mRNA-LNP improves Glut-4 expression *in situ*, leading to an improvement in glucose uptake.

### Improvement of the Insulin resistant pathway (DGKd and PKCε)

#### DGKd

*In vitro*: The results showed a significant downregulation of DGKd gene expression in the untreated group (Empty-LNP) as compared with the NC group. However, after APN-mRNA-LNP transfection for 24, 48, and 72 h, DGKd gene expression significantly increased in C2C12 myotubes at all timepoints and in SV40-MES13 kidney mesangial cells when compared with the PC and Empty-LNP groups.

*In vivo*: The results showed a significant upregulation of DGKd gene expression in skeletal muscles, liver, and kidney after 3 days, 1 week, and 2 weeks of APN-mRNA-LNP injections as compared with the PBS and Empty-LNP groups (Fig. 3B).

Regarding DGKd protein expression, the results showed a significant reduction in the PC and Empty-LNP groups (p < 0.001) when compared with the NC group. However, when the treated groups were compared witn the PC and Empty-LNP groups, DGKd protein expression significantly increased after 24, 48, and 72 h of transfection in C2C12 myotubes (p < 0.001, 0.001, and 0.01, respectively) and in SV40-MES13 kidney mesangial cells, exhibiting a significant gradual increase (p < 0.001) at all timepoints (Fig. 3D, E). This indicates that the injection of APN-mRNA-LNP results in an increase in DGKd expression in the diabetic mouse model.

#### PKCε:

*In vitro*: The results showed a significant upregulation of PKCε gene expression in the untreated group (PBS and Empty-LNP) as compared with the NC group. However, after APN-mRNA-LNP transfection for 24, 48, and 72 h, PKCε gene expression significantly decreased in C2C12 myotubes at all timepoints and in SV40-MES13 kidney mesangial cells when compared with the PC and Empty-LNP groups.

*In vivo*: The findings indicated a significant downregulation of PKCε gene expression in skeletal muscles, liver, kidney, W. fat, and B. fat tissues after 3 days, 1 week, and 2 weeks of APN-mRNA-LNP injections as compared with the PBS and Empty-LNP groups, as illustrated in (Fig. 3C).

In terms of PKCε protein expression, the PC and Empty-LNP groups showed a significant increase (p < 0.001) as compared with the NC group. However, after 24, 48, and 72 h of transfection in C2C12 myotubes (p < 0.001 at all timepoints) and in SV40-MES13 kidney mesangial cells, PKCε protein expression significantly and gradually decreased (p < 0.001) at all timepoints (Figure 3D, E) in the treated groups as compared with the PC and Empty-LNP groups (Fig. 3D, E). These results indicate that the injection of APN-mRNA-LNP results in the inhibition of PKCε activation in the diabetic mouse model.

**Figure 4.**
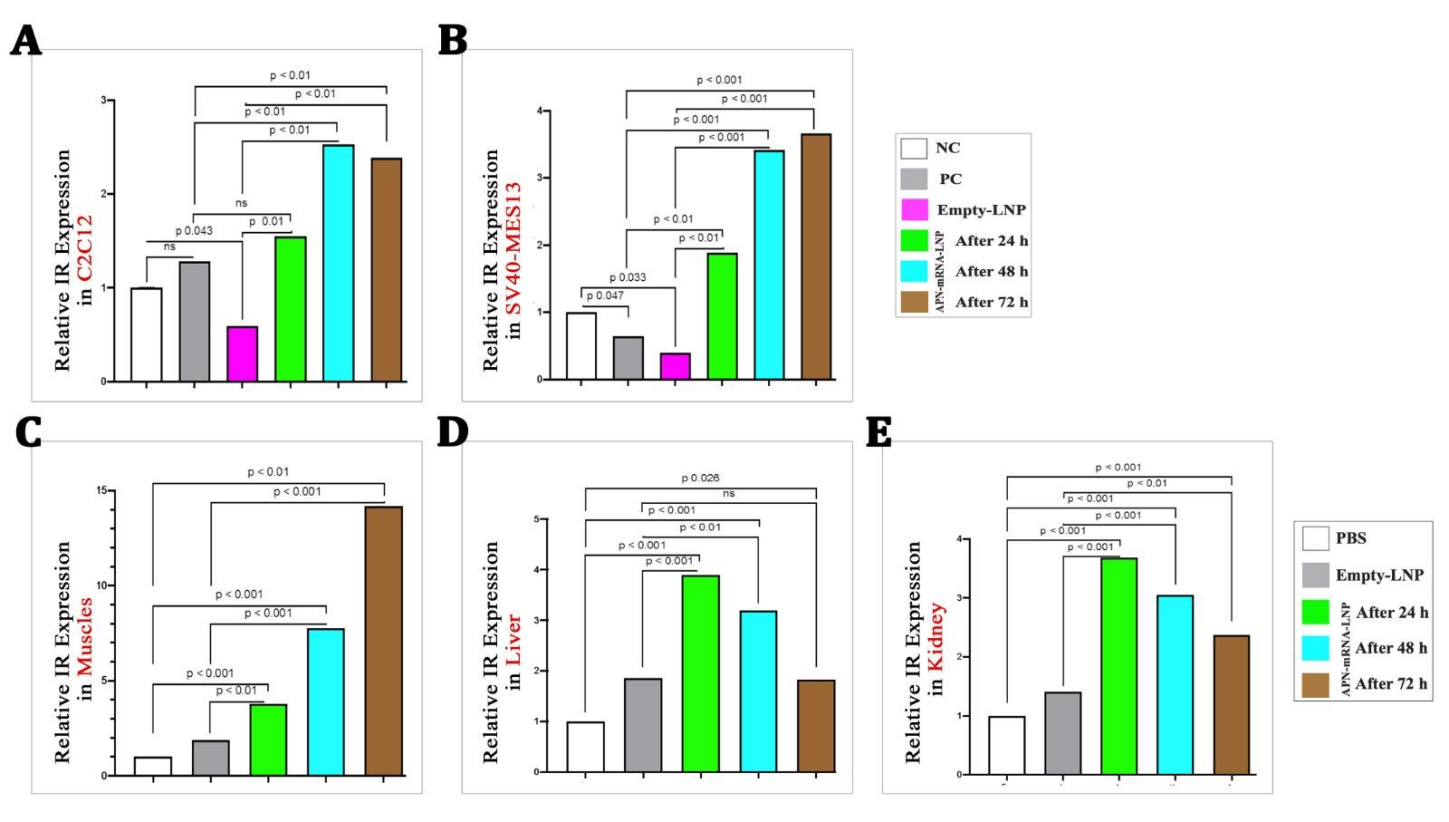
**Relative IR expression in different studied tissues**. (**A**) Differentiated Myotubes cell C2C12; (B) Kidney mesangial cell SV40-MES13; (C) Skeletal muscles; (D) Liver; (E) Kidney. IR gene expression was calculated based on the Law of Fold-change, which is 2^-ΔΔCT^. Fold-change values less than one indicate downregulation, whereas values greater than one indicate upregulation. Both biological and technical triplicates were performed *in vitro*. Values are mean ± SD. n = 9, student t-test. *In vivo*, a total of five biological samples (n = 5, student t-test) were collected. Of each sample, three replicates were performed technically. Significances are shown above bars.

### Insulin receptor (IR) activation

*In vitro*: The results showed a significant downregulation of IR gene expression in the untreated group (Empty-LNP) as compared with the NC group. However, after APN-mRNA-LNP transfection for 24, 48, and 72 h, IR gene expression significantly increased in C2C12 myotubes in all timepoints (Fig. 4A), as well as in SV40-MES13 kidney mesangial cells (Fig. 4B), when compared with the PC and Empty-LNP groups. Thus, the injection of APN-mRNA-LNP results in IR activation in the diabetic mouse model.

*In vivo:* The results showed a gradual and significant upregulation of IR gene expression in skeletal muscles (Fig. 4C). In liver and kidney, the levels increased significantly after 3 days and 1 week, but decreased after 2 weeks of APN mRNA-LNP injections as compared with the PBS and Empty-LNP groups (as shown in Fig. 4D, E). This indicates that injection of APN-mRNA-LNP results in IR activation in the diabetic mouse model.

**Figure 5.**
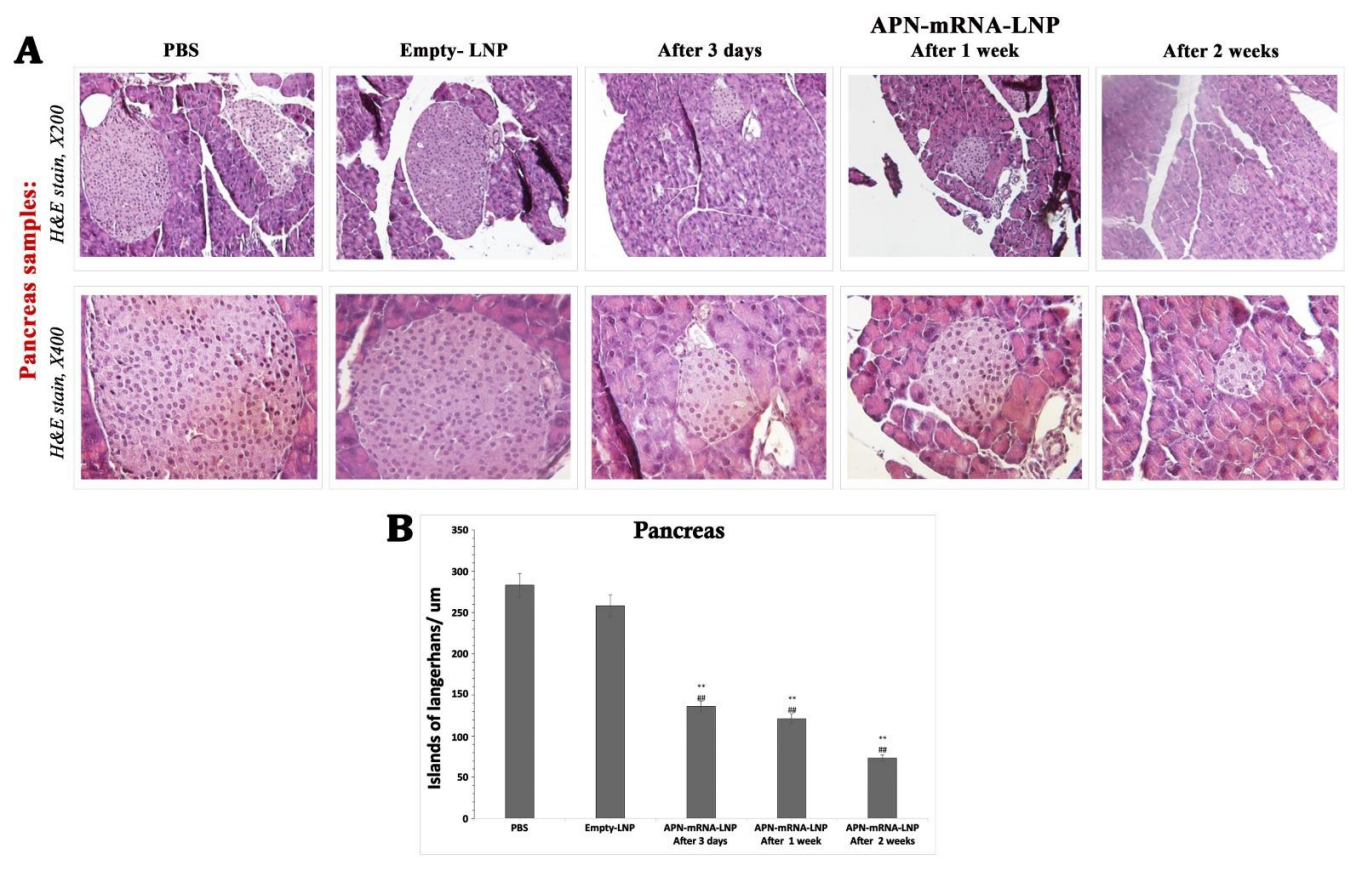
**Pancreas sections in the studied groups**. (**A**) Pancreas sections focusing on islands of *Langerhans* diameter (H&E stain, 200X & 400X). (**B**) Islands of *Langerhans* average diameter; the diameter average was taken from seven fields on the same slid (n = 7, student t-test), Significances are shown above the bars: ^**^P < 0.01 vs. the PBS and ^##^P < 0.01 vs. Empty-LNP. Scale bar= 50 μm.

### Insulin secretion activation through the reactivation of the pancreatic islets

To assess the correlation between APN overexpression and the improvement of insulin secretion within the islets of Langerhans, pancreas specimens were collected and fixed in a 10% buffered formaldehyde solution for 24 h for subsequent histological examinations. A comparison of the islet diameter before and after injection at various time points was conducted. The results showed that pancreatic tissue sections from the PBS and Empty-LNP groups exhibited large-sized islets of Langerhans with average diameters of 283 and 258.3 μM, respectively. On the other hand, the diameter slightly decreased in the APN-mRNA-LNP group after three days (136 μM) and one week (121.25 μM). Notably, the diameter of the islets of Langerhans significantly decreased in the APN-mRNA-LNP group after two weeks, reaching 73.33 μM (Fig. 5A, B).

### EGFR pathway inhibition

The fifth factor in our research focuses on EGFR activity, which directly contributes to insulin resistance and diabetic nephropathy. Therefore, we sought to confirm the relationship between APN overexpression and the inhibition of EGFR activity.

**Figure 6.**
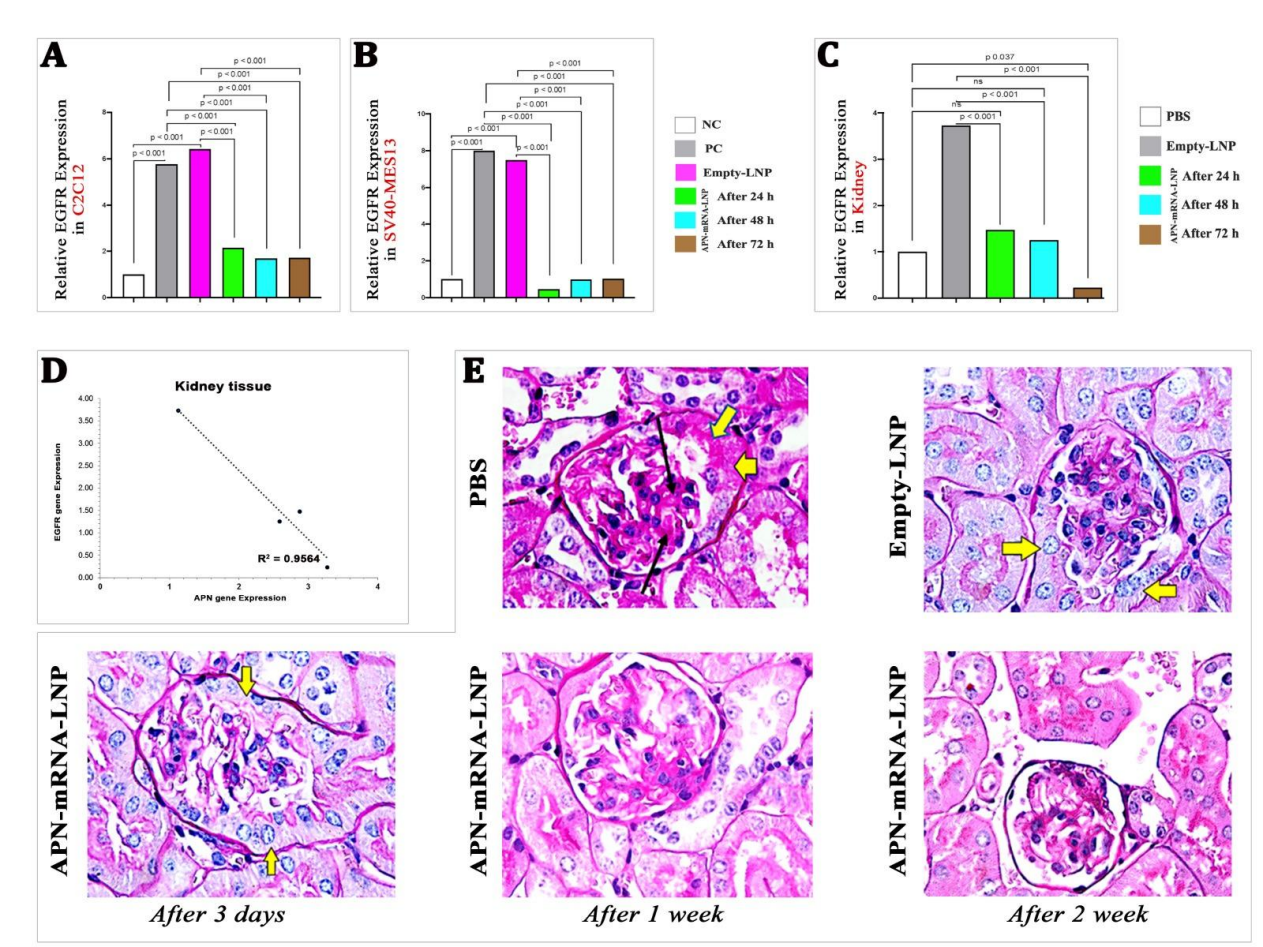
**EGFR expression *in vitro* and *in vivo***. (A) Relative EGFR Expression in different Differentiated Myotubes cell C2C12; (B) Kidney mesangial cell SV40-MES13; (C) kidney. EGFR gene expression was calculated based on the Law of Fold-change, which is 2^-ΔΔCT^. Fold-change values less than one indicate downregulation, whereas values greater than one indicate upregulation. Both biological and technical triplicates were performed *in vitro*. Values are mean ± SD. n = 9, student t-test. *In vivo*, a total of five biological samples (n = 5, student t-test) were collected. Of each sample, three replicates were performed technically. Significances are shown above bars. (**D**) Overall correlation between APN and EGFR gene expression in kidney. (**E**) Light microscopic appearance in kidney tissue section of the studied groups of DIO mice, (yellow arrow) indicates Hyperplasia of partial cells in the Bowmans capsule of the glomeruli and (Black arrow) indicates mild diffuse mesangial matrix expansion (PAS stain, 1000X). A total of five biological samples (n = 5, student t-test) were collected; three sections were taken from each sample. Scale bar= 50 μm.

*In vitro*: The results showed a significant upregulation of EGFR gene expression in the untreated group (PBS and Empty-LNP) as compared with the NC group. However, after APN-mRNA-LNP transfection for 24, 48, and 72 h, EGFR gene expression significantly decreased in C2C12 myotubes at all timepoints (Fig. 6A), as well as in SV40-MES13 kidney mesangial cells (Fig. 6B), when compared with the PC and Empty-LNP groups.

*In vivo*: The findings showed a significant decrease in EGFR gene expression in the kidney after 3 days and 1 week of APN-mRNA-LNP injection as compared with the Empty-LNP groups. Remarkably, there was a significant further decrease after two weeks of injection as compared with the PBS and Empty-LNP groups (Fig. 6C).

Additionally, the overall correlation study between APN and EGFR expression in kidney tissue showed a significantly negative correlation (r = -0.978, p = 0.022) (Fig. 6D), indicating that the injection of APN-mRNA-LNP leads to EGFR inhibition in the diabetic mouse model.

The evidence of the relationship between APN overexpression and EGFR inhibition prompted investigation into the potential impact of APN-mRNA-LNP injections on other pathological conditions related to diabetic nephropathy. As a result, kidney specimens were collected, fixed in a 10% buffered formaldehyde solution for 24 h, and then cut into 3 μM-thick sections for PAS staining. Pathological findings showed that, in both the treated and untreated groups (PBS and Empty-LNP), there was persistent hyperplasia of partial cells in the Bowman's capsule of the glomeruli, even after three days. However, in most of the fields displayed, hyperplasia started to decrease after 1 week of injection and completely disappeared after 2 weeks (Fig. 6E; yellow arrows). Furthermore, mild mesangial matrix expansion diffusion was observed in the PBS and Empty-LNP groups but completely disappeared in the treated groups at different time points (Fig. 6E; black arrows).

### Inhibition of Inflammatory cytokine production

Proinflammatory cytokines have been found to decrease with diabetes treatment, and EGFR inhibition has also been demonstrated to reduce the production of these cytokines (46). Therefore, we sought to verify this relationship by examining the common proinflammatory cytokines directly related to T2D (TNF-α, IL-6, and IL-1b).

**Figure 7.**
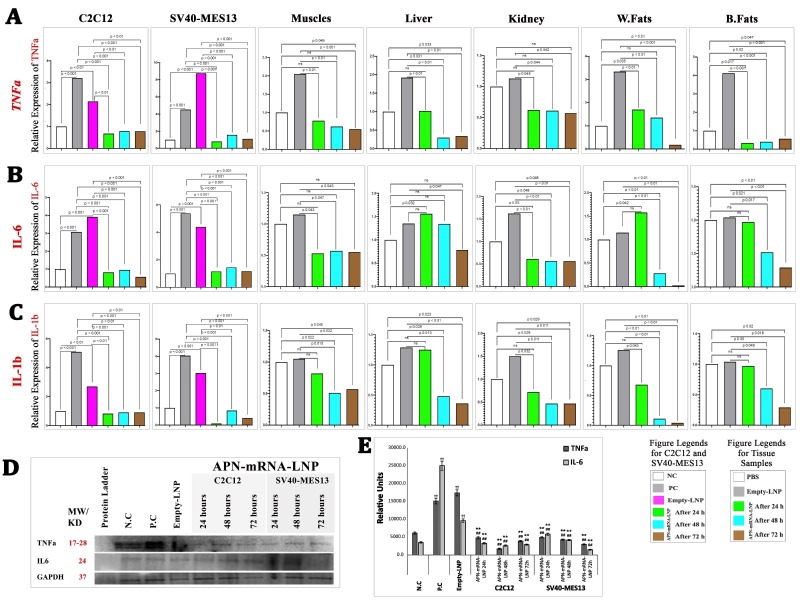
**Relative gene expression of the pro-inflammatory cytokines**. (**A**) TNFa gene in differentiated myotubes cell C2C12, Kidney mesangial cell SV40-MES13, Skeletal muscles, Liver, Kidney, White fats, and Brown fats. (**B**) IL-6 gene in differentiated myotubes cell C2C12, Kidney mesangial cell SV40-MES13, Skeletal muscles, Liver, Kidney, White fats, and Brown fats. (**C**) IL-1b gene in differentiated myotubes cell C2C12, Kidney mesangial cell SV40-MES13, Skeletal muscles, Liver, Kidney, White fats, and Brown fats. Gene expression was calculated based on the Law of Fold-change, which is 2^-ΔΔCT^. Both biological and technical triplicates were performed *in vitro*. Values are mean ± SD. n = 9, student t-test. *In vivo*, a total of five biological samples (n = 5) were collected. Of each sample, three replicates were performed technically. (**D**) Glut-4 TNFa and IL-6 protein expression, three separate blot analysis of the samples were conducted. (**E**) Optical densitometry was used to semi-quantify the bands, and ImageJ (1.53t) digital imaging processing software was used for analysis. GAPDH has been used as an endogenous control for protein expression. Protein levels are represented as mean ratio values quantified from protein bands of the targeted protein versus GAPDH compared to negative control. (n = 3, Mann-Whitney U tests), Significances are shown above bars: ^††^P < 0.01 vs. negative control (NC), ^**^P < 0.01 vs. positive control (PC) “PBS” and ^##^P < 0.01 vs. Empty-LNP.

*In vitro*: Results showed that TNF-α, IL-6, and IL1b gene expression were significantly upregulated in the untreated group (PBS and Empty-LNP) as compared with the NC group. However, gene expression in SV40-MES13 renal mesangial cells and C2C12 myotubes decreased significantly at all timepoints following APN-mRNA-LNP transfection for 24, 48, and 72 h (Fig. 7A, B, and C), respectively.

*In vivo*: Results showed that TNF-α gene expression was significantly downregulated in skeletal muscles, liver, W. fats, and B. fat tissues after 1 week and 2 weeks of APN-mRNA-LNP injections as compared with the PBS and Empty-LNP groups. Marginally significant differences were observed at different time points in the kidney when compared with the Empty-LNP groups (Fig. 7). After receiving APN-mRNA-LNP injections for 1 week and 2 weeks, the kidney, W. fat, and B. fat tissues showed a significant downregulation of IL-6 gene expression as compared with the PBS and Empty-LNP groups. Slightly significant differences were observed at different time points in the skeletal muscles, while a significant difference was noted in the liver after 2 weeks of injections. Following 1 week and 2 weeks of APN-mRNA-LNP injections, IL-1b gene expression was significantly reduced in skeletal muscles, liver, kidney, W. fat, and B. fat tissues as compared with the PBS and Empty-LNP groups (Fig. 7A, B, and C).

**Figure 8.**
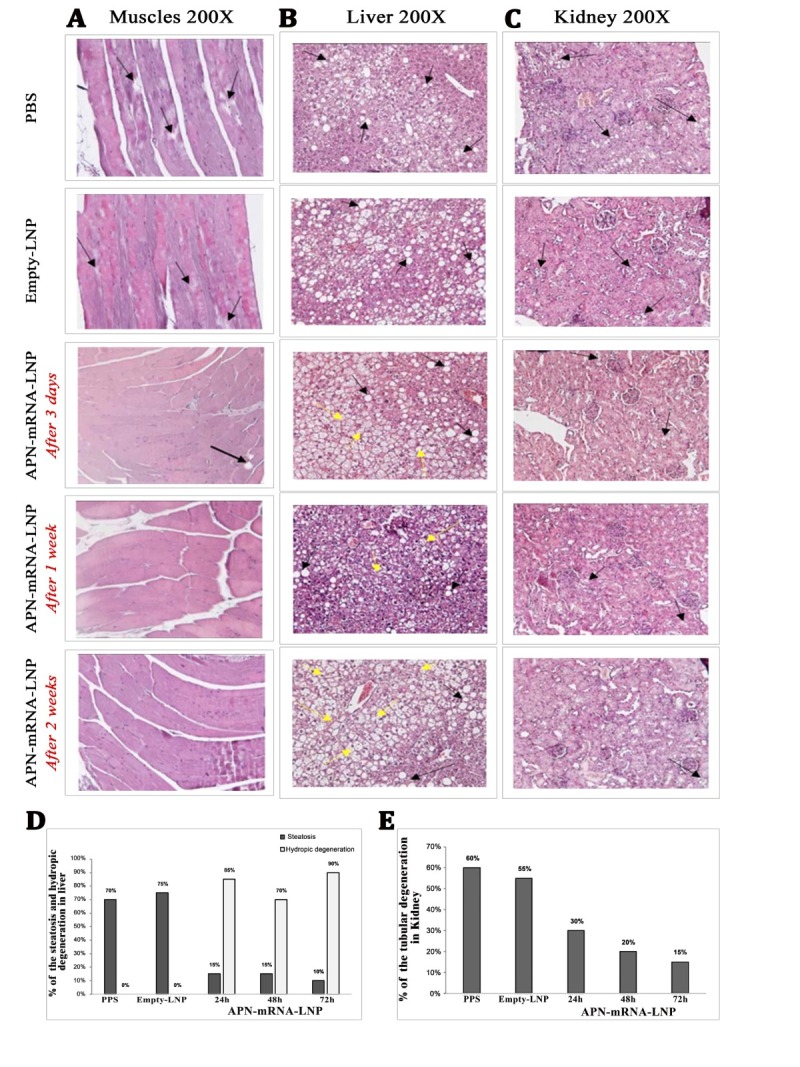
**Histopathology results in the studied groups**. (**A**) longitudinal section of the skeletal muscles of the femur, ranks score were used to represent the marked focal degeneration of the skeletal muscle bundles, which signed with different amount of “+” depending on how many other categories lay between these border parameters “weak” (+), “moderate” (++), “strong” (+++) and their variations. (**B**) liver samples; (C) light microscopic appearance in the kidney tissue section (H&E stain, 200X); black arrows indicate fat degenerations, while yellow arrows indicate hydropic degenerations. (**D**) Percentile of steatosis and hydropic degeneration in the liver of the studied mouse groups; (E) Percentile of tubular degeneration in the kidney of the studied mouse groups. The percent of fat degeneration in different samples and/or hydropic degeneration was calculated depending on Labeling index (the ratio of positively stained cells/total cells×100). A total of five biological samples (n = 5, student t-test) were collected; three sections were taken from each sample. Scale bar= 50 μm.

In terms of TNF-α and IL-6 protein expression, the PC and Empty-LNP groups showed considerably higher levels (p < 0.001) than the NC group. TNF-α and IL-6 protein expression in the treated groups was significantly lower than that of the PC and Empty-LNP groups after 24, 48, and 72 h of transfection, similar to the normal negative control group in C2C12 myotubes (p < 0.001) at all timepoints, as well as in SV40-MES13 kidney mesangial cells (p < 0.001) at all timepoints (Fig. 7D, E). This suggests that the injection of APN-mRNA-LNP leads to the inhibition of inflammatory cytokine activation in the diabetic mouse model.

### Fatty changes reduction

The invasion of adipose tissue is pathologically associated with obesity, insulin resistance, and diabetes through the generation of cytokines and chemokines by immune cells (B cells and T cells) and macrophages [55]. One of the negative consequences of insulin resistance in T2D is the breakdown of fat in the muscles, liver, and kidneys, which in severe cases, can spread to the brain. Studies have shown a correlation between the down-regulation of fat degeneration (hydropic degeneration) and APN overexpression. Skeletal muscles from the femur, liver, and kidney samples were used to evaluate this relationship. Pathological findings showed that in the longitudinal sections (LS) of the skeletal muscle tissue of mice in the BPS and LNP-Empty groups, the focal degeneration of the skeletal muscle bundles was marked with (++) (black arrow). In APN-mRNA-LNP, focal degeneration at 3 days was slightly less than in the previous groups (+). It is worth note that the focal degeneration of the skeletal muscle bundle was almost absent in APN-mRNA-LNP after 1 week and 2 weeks (Fig. 8A).

Sections of liver tissue from PBS and Empty-LNP showed almost identical features and percentages of clearly distributed hepatocellular fatty changes (steatosis) in approximately 60-80% and 70-80% of hepatocytes, respectively (black arrow). On the other hand, sections of liver tissue from the APN-mRNA-LNP groups after 3 days, 1 week, or 2 weeks showed a significant reduction in hepatocellular steatosis, affecting almost 10-20%, 10-20%, and 5-10% of cells in a descending order, respectively. Interestingly, the proportion of hepatocellular hydropic degeneration appeared simultaneously with intervals of 80-90%, 60-80%, and 90-100%, respectively, following 3 days, 1 week, and 2 weeks of injection (Fig. 8B, D).

Light microscopy of kidney tissue sections from PBS and Empty-LNP mice showed almost identical features, with an average of 60-70% and 50-60%, respectively (black arrow). Sections of kidney tissue from the APN-mRNA-LNP groups after 3 days, 1 week, or 2 weeks had a significant decrease in tubular degeneration, with tubular cells in a descending route: 30-35%, 15-25%, and 10-15%, respectively (Fig. 8C, E).

## DISCUSSION

T2D is characterized by a malfunction in the body's capacity to regulate and utilize glucose as an energy source. This chronic condition results in elevated blood sugar levels. Over time, high blood sugar can lead to complications affecting various systems and major organs including the liver, kidney, heart, and brain. There are two main issues associated with T2D: insufficient insulin production by the pancreas, and reduced sensitivity of cells to insulin, leading to poor sugar uptake. As a result, the present study was conducted to address these issues by promoting *in situ* production of adiponectin, APN, a protein closely involved in the insulin resistance pathways. Through the application of APN-mRNA-LNP, we successfully increased the direct production of insulin, boosted the uptake of glucose into cells, and decreased inflammation associated with uncontrolled hyperglycemia.

The results of the present study showed that the APN-mRNA-LNP treatment resulted in a significant increase in the expression of both APN protein and gene, both *in vitro* and *in vivo*.

Moreover, this treatment had a direct impact on reducing body weight and blood glucose levels. To function in vivo, for mRNAs function *in vivo*, it requires reliable, efficient, and robust delivery mechanisms that protect nucleic acids from degradation and allow cellular uptake and mRNA release. The stability and safety of employing APN-mRNA-LNP in both *in vivo* and *in vitro* settings were supported by our results. These findings are in line with previous study [35] and highlighted the potential of APN mRNA-LNP as a novel therapeutic agent to prevent and treat various diseases.

The present study evaluated the Glut-4 gene expression at both gene or protein level in the C2C12 myotubes and SV40-MES13 kidney mesangial cells, as well as in the skeletal muscles, liver, kidney, W. fat, and B. fat tissues. Significantly elevated levels of Glut-4 expression were observed in these tissues. Consequently, an improvement in glucose uptake was observed. These results are in lined with previous findings that globular adiponectin enhances glucose absorption in skeletal muscle cells via GLUT4 translocation, which slows the rate of glycogen formation and causes a shift in glucose metabolism toward lactate generation [56]. These effects align with the enhanced AMP kinase, acetyl-CoA carboxylase phosphorylation, and fatty acid oxidation brought on by globular adiponectin.

In our mechanistic study, after injection with APN-mRNA-LNP, the DGKd was significantly upregulated in C2C12 and SV40-MES13 cell lines, as well as in the skeletal muscles, the liver, and the kidney of DIO mice. The preceding relationship is in line with similar studies [57-59] that have shown that ectopic lipids are associated with insulin resistance in liver and skeletal muscle. Improvements in liver and muscle sensitivity to adiponectin were associated with a 50% reduction in TAG and PM-associated DAG content in liver and muscle [60]. As a precursor of triglycerides and phospholipids, DAG contributes to the metabolism of lipids, and functions as a second messenger in cellular signaling. A temporal increase in intracellular DAG mass is associated with glucose-induced insulin resistance in animals [39]. DAG is also phosphorylated to produce phosphatidic acid (PA), which is needed for it to act as a lipid second messenger and regulate signals related to metabolic and mitogenic responses [61]. A disruption in the equilibrium between intracellular DAG and PA levels might harm cellular metabolism. DGKd is a class of enzymes important for reducing DAG signaling and catalyzing the phosphorylation of DAG to PA [62]. Peripheral insulin resistance and moderate obesity are implicated in decreased DGKd protein expression. Skeletal muscle DGKd is downregulated in those with poorly managed blood sugar levels [43].

A persistent upturn in intracellular DAG promotes aberrant signal transduction and intracellular lipid accumulation through the initiation of PKC isoforms and insulin resistance [40, 41]. In the present study, DGKd showed significantly downregulated gene and/or protein expression in C2C12, SV40-MES13 cells, even in the skeletal muscles, liver, kidney, W. fat, and B. fat tissues of DIO mice after being injected with APN-mRNA-LNP. This indicates that APN-mRNA inhibits PKCε activity. Li et al. [61] report that the amounts of sn-1,2-DAG associated with the plasma membranes of the liver and muscles decreased due to adiponectin activation. This resulted in decreased PKCε activity in the liver and PKCε and PKCθ activity in the muscles. In these tissues, insulin signaling, and action were heightened as a result.

Decreased PM DAG content in the organs was linked to reduced PKCε translocation in the liver and reduced PKCε and PKCθ translocation in the skeletal muscles. This resulted in an increase in insulin-stimulated insulin receptor tyrosine1162 phosphorylation, IRS-1/IRS-2-associated PI3-kinase activity, and Akt-serine phosphorylation [60]. Based on this mechanism, the results of the present study showed a significant increase in IR gene after injection of APN-mRNA-LNP in DIO mouse’s skeletal muscle, liver, kidney, and C2C12 and SV40-MES13 cell lines.

The present study showed that APN-mRNA-LNP injection significantly reduced the diameter of the islets of *Langerhans* in the pancreas of DIO mice. It is well known that diet-induced mouse islet hyperplasia is often linked to the persistent elevation of insulin levels, a risk factor for T2D and metabolic disorders. This phenomenon is characterized by a compensatory expansion of the functional mass of beta-cells, notably observed in individuals with obesity [63] Our study showed that APN-mRNA-LNP administration successfully reduced islet size in HFD-fed mice, which is associated with enhancement of insulin secretion. This evidence supports our finding that APN-mRNA-LNP administration improve diet-induced dys-regulation of pancreas function by averting islet hyperplasia.

While the diameter in the untreated groups (BPS and Empty-LNP) showed a huge size (283 and 258.3 μM, respectively), it decreased after two weeks of injection to 73.33 μM, as previously reported; meanwhile, the mature normal pancreas of mice contains numerous islets of *Langerhans*, which are held up by a mass of branching exocrine tissue. While the observed reduction in islet size might seem counterintuitive, it could potentially improve beta cell function by enhancing cell-to-cell contact and facilitating blood flow within the islets. Future studies employing immunocytochemical analysis of beta cell mass would be valuable to further explore this aspect. In addition, the apparent shrinkage of the islet is beneficial as it loses penetration of exocrine tissue [64]. The islets vary greatly in size; on average, they have a diameter of between 50 and 200 μM. Insulin secretion is activated in DIO mice injected with APN-mRNA-LNP, based on the concept that island size and insulin production are correlated irreversibly [65].

Following APN-mRNA-LNP injection, the expression of the EGFR gene was significantly downregulated in the kidneys of DIO mice, as well as in the C2C12 and SV40-MES13 cell lines. It is worthy of note that a clear negative correlation was observed between APN and EGFR gene expression in the kidney (r = -0.978, p = 0.022) (Fig. 6D). Moreover, unexpected results were observed, as certain features of the moderate mesangial matrix expansion diffusion and hyperplasia of cells in the *Bowman's* capsule of the glomeruli appeared to reduce in the APN-mRNA-LNP injected groups when compared with the untreated groups. These indicate that the administration of APN-mRNA-LNP results in the inhibition of EGFR, which is consistent with the pathway proposed by Li et al. [44]. In their study, they used both pharmacological and genetic EGFR inhibition to investigate the possible role of EGFR activation to the development of DN in the eNOS/db/db model of accelerated T2D. The key conclusions from their study include: 1) Erlotinib's ability to inhibit the activity of the EGFR tyrosine kinase slowed the course of DN; 2) Erlotinib reduced oxidative stress and renal macrophage and lymphocyte infiltration; 3) Erlotinib therapy increased glucose tolerance, insulin sensitivity, and pancreatic insulin expression while decreasing body weight growth, adipose tissue mass, and fasting blood sugar; 4) Adiponectin was shown to be more abundant in the blood after taking erlotinib; and 5) lower F2-isoprostanes in the urine showed that erlotinib reduced systemic oxidative stress [44]. These findings provide additional support for the potential therapeutic effects of APN-mRNA-LNP and its impact on EGFR activity in the context of diabetes-related complications such as DN.

The expression of pro-inflammatory cytokines is reduced by EGFR inhibition [46]. Based on the results of the present study, the C2C12 and SV40-MES13 cell lines showed significantly reduced gene and protein expression of TNF-α and IL-6, as well as IL-1b gene expression, following APN-mRNA-LNP transfection at different time periods. However, TNF-α gene expression was shown to be significantly reduced in skeletal muscles, liver, W. fat, and B. fat tissues, but not in the kidney, following 1 and 2 weeks of APN-mRNA-LNP injection. These *in vivo* results were more selective. Similarly, IL-6 levels significantly decreased in the kidney, W. fat, and B. fat tissues after 1 and 2 weeks of APN-mRNA-LNP injection, while no significant changes were observed in skeletal muscles and liver IL-6 levels. Notably, following 1 and 2 weeks of APN-mRNA-LNP injection, the expression of the IL-1b gene significantly decreased in all examined organs, including skeletal muscles, the liver, kidney, W. fat, and B. fat tissues. Therefore, it can be said that APN-mRNA-LNP treatment resulted in a reduction of pro-inflammatory cytokines (IL-1b, IL-6, and TNF-α). Precisely, most of the organs were studied *in vivo* after two weeks, suggesting that the lack of decrease in some organs after this period may be attributed to changes in the organ's response to reduced inflammation. Our results indicate that the use of APN-mRNA compensates for the negative effects of LNPs on cytokines, which is a drawback of LNPs. These results are in line with earlier studies that pro-inflammatory cytokine production was decreased in adiponectin-knockout animals following renal ischemia-reperfusion [66]. The accumulated W. fat, especially around the trunk, upper body or abdomen, appears to be the main source of inflammatory indicators in T2D, and it also serves as a platform for the development of inflammation in individuals with diabetes. It produces cytokines and a wide range of other bioactive substances involved in the inflammatory pathways, including TNF-α, IL-1, IL-6, IL-10, chemokines, serum amyloid protein, leptin, adiponectin, resistin, and many other substances generally referred to as adipokines [67-70]. Obesity, insulin resistance, and diabetes are pathologically associated with the production of several cytokines and chemokines due to increased infiltration of macrophages and immune cells (B cells and T cells) into adipose tissue [55].

In the present study, histopathological examination of skeletal muscle, liver, and kidney in treated DIO mice showed significant recovery from fatty degeneration 3 days and 1 or 2 weeks after APN-mRNA-LNP injection. The results suggest that APN-mRNA has positive benefits in reducing inflammation associated with metabolic disorders caused by lipid accumulation and insulin resistance. These results are in line with reports that interrelated processes of lipotoxicity and glucotoxicity result in IR impairment and reduced insulin secretion [66]. Furthermore, ATP-citrate lyase (ACL), fatty acid synthase (FAS), and stearoyl-CoA desaturase (SCD)-1 are genes that are activated by chronically elevated plasma glucose levels via two different mechanisms: directly, by increasing the citric acid (TCA) cycle activity and synthesis of Acyl CoA, which serves as a substrate for both gluconeogenesis and de novo lipogenesis (DNL) [71]; and indirectly, by activating the expression of carbohydrate response element binding protein (ChREBP) and liver X receptor α (LXRα), which in turn promotes ACL, FAS, and SCD-1 gene transcription [72]. Moreover, glucotoxicity increases lipotoxicity and activates ChREBP in the kidney, skeletal muscles, and pancreas, decreasing insulin production (pancreas) and aggravating IR in these tissues. ChREBP expression is decreased in adipocytes by regulating the release of specific adipokines and lipid species, which may exacerbate the condition of IR [72]. To verify its influence across a variety of broad dimensions, we recommend conducting single cell sequencing on DIO mice with a longer follow-up period beyond the initial 2 weeks following injection with APN-mRNA-LNP. To confirm its impact on a wide range of dimensions, we recommend single-cell sequencing on DIO mice with a longer follow-up period beyond the initial 2 weeks following administration of APN-mRNA-LNP. This will provide a more comprehensive understanding of the impact of the treatment across various dimensions.

**Figure 9.**
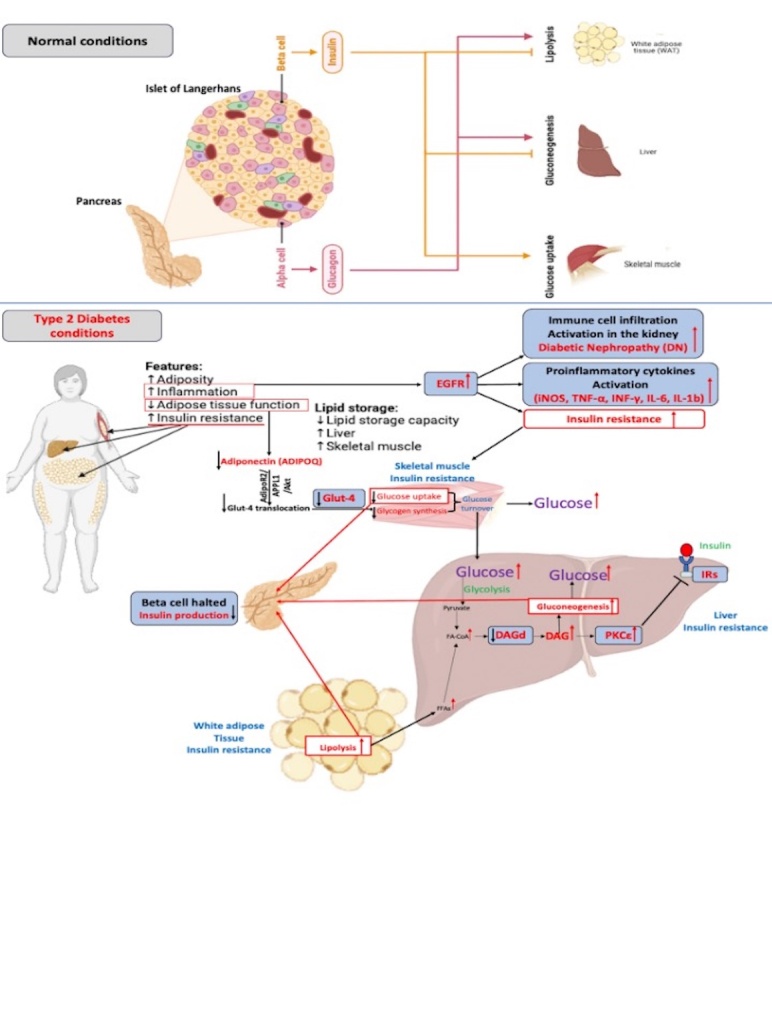
**Conclusion based on our hypothesis**. The alpha and beta cells of the islet of Langerhans work together to control and regulate glucose uptake, gluconeogenesis, and lipolysis in normal conditions. There are several abnormal features associated with type 2 diabetes, including increased adiposity, inflammation, insulin resistance, and decreased adipose tissue function. Thus, adiponectin (APN) production is reduced. As a result of the inhibition of adiponectin receptors (AdipoR1/AdipoR2) and subsequent association with adapter proteins, glucose uptake is inhibited by APPL1-Rab5 or APPL1-AMP-AMPK-mediated translocation of glucose transporter 4 (GLUT4), resulting in an activation of insulin resistance signaling [36]. Insulin resistance is commonly characterized by decreased GLUT4-dependent glucose absorption in skeletal muscles and adipose tissue [38]. A temporal increase in intracellular diacylglycerol (DAG) mass is associated with glucose-induced insulin resistance [39]. Prolonged elevation of intracellular DAG activates protein kinase C (PKC) isoforms, leading to insulin resistance, intracellular lipid accumulation, and impaired signal transmission [40, 41]. Glucose transport is downregulated due to increased PKC-mediated serine phosphorylation of the insulin receptor (IR) and insulin receptor substrate 1 (IRS-1) [42, 43]. Controlling hyperglycemia can reverse the decline in diacylglycerol kinase delta (DGKd) protein and DGK kinase activity [43]. Moreover, insulin resistance and diabetic nephropathy (DN) in T2D are associated with epidermal growth factor receptor (EGFR) activation. This activation increases immune cell infiltration and oxidative stress in the kidney and adipose mass while simultaneously reducing pancreatic insulin synthesis and adipocyte adiponectin production [44]. Inhibition of EGFR decreases the expression of proinflammatory cytokines (iNOS, TNF-α, INF-γ, IL-6) [46]. As a result of the administration of APN-mRNA-LNP, we have found that all previous targets have been successfully corrected. The result was improvements in several aspects of diabetes pathogenesis, including glucose uptake, insulin resistance, inflammation, and diabetic complications.

The results of the present study show that APN-mRNA-LNP stimulates the formation of endogenous APN in muscles, liver, kidney, pancreas, and fat cells. APN-mRNA-LNP, after being administered, was proven to be safe for experimental animals because it quickly disappears from the body and does not integrate into the genome. As a result of the improvement in APN, 1) glucose uptake was improved through Glut-4 activation. 2) Insulin resistance was attenuated by DGKd activation, which was related to DAG and PKCε inhibition. 3) Insulin receptor activation by inhibiting the PKCε. 4) Reactivation of *Langerhans* islands to stimulate insulin secretion. 5) EGFR pathway blocking, which results in the alleviation of DN markers. 6) Pro-inflammatory cytokines inhibition (TNF-α, IL-Ib, IL-6), leading to inflammation reduction. 7) Alleviation of fat accumulation in muscles, liver, and kidney. All this led to a reduction in body weight and an improvement in glucose synthesis (Fig. 9).

In conclusion, our findings indicate that APN-mRNA-LNP effectively addresses multiple aspects of T2D pathogenesis, including glucose uptake, insulin resistance, inflammation, and diabetic complications. The mRNA-LNP-based nucleic acid therapy emerges as a promising and efficient approach for targeting the underlying mechanisms of T2D.

### Future Studies

We propose two key enhancements to our study. First, we aim to implement a more comprehensive follow-up plan to evaluate the enduring effects and stability of APN-mRNA-LNP treatment over the long term. Second, we intend to conduct an in-depth exploration of the molecular mechanisms through which APN-mRNA-LNP influences various pathways associated with T2D. Our forthcoming investigations will overcome these limitations by employing extended follow-up protocols and utilizing bulk RNA-seq and/or single-cell RNA-seq methodologies for a thorough examination of the molecular cascades post-APN-mRNA-LNP administration. Furthermore, in the advanced stages of our research, we plan to incorporate the following measures: inclusion of additional control groups receiving established T2D therapies, exploration of a broader range of dosages, augmentation of sample size, and the incorporation of a more diverse participant pool in terms of age, gender, and ethnicity. We also aim to conduct a meticulous assessment of potential side effects and the long-term safety profile of APN-mRNA-LNP treatment, as well as investigate potential interactions between APN-mRNA-LNP and commonly prescribed medications and prevalent medical conditions.

## Supplementary Materials

The Supplementary data can be found online at: www.aginganddisease.org/EN/10.14336/AD.2024.0162.


