# Supplementary material for: Adiponectin mRNA Conjugated with Lipid Nanoparticles Specifically Targets the Pathogenesis of Type 2 Diabetes
Source: Aging Dis. 2024 May 15;16(2):1059–79. doi: 10.14336/AD.2024.0162 (PMC11964417; doi:10.14336/AD.2024.0162)
Supplement: Supplementary file 1 [file AD-16-2-1059-s.pdf]

SUPPLEMENTARY DATA

Adiponectin mRNA Conjugated with Lipid Nanoparticles Specifically Targets the Pathogenesis of Type 2 Diabetes

Rady E. El-Araby, Qisheng Tu, Ying Xie, Tarek Aboushousha, Zhongyu Li, Xiaoyang Xu, Zoe X. Zhu, Lily Q. Dong, Jake Chen

© 2024. El-Araby RE et al. Published online at http://www.aginganddisease.org/EN/10.14336/AD.2024.0162

SUPPLEMENTARY DATA

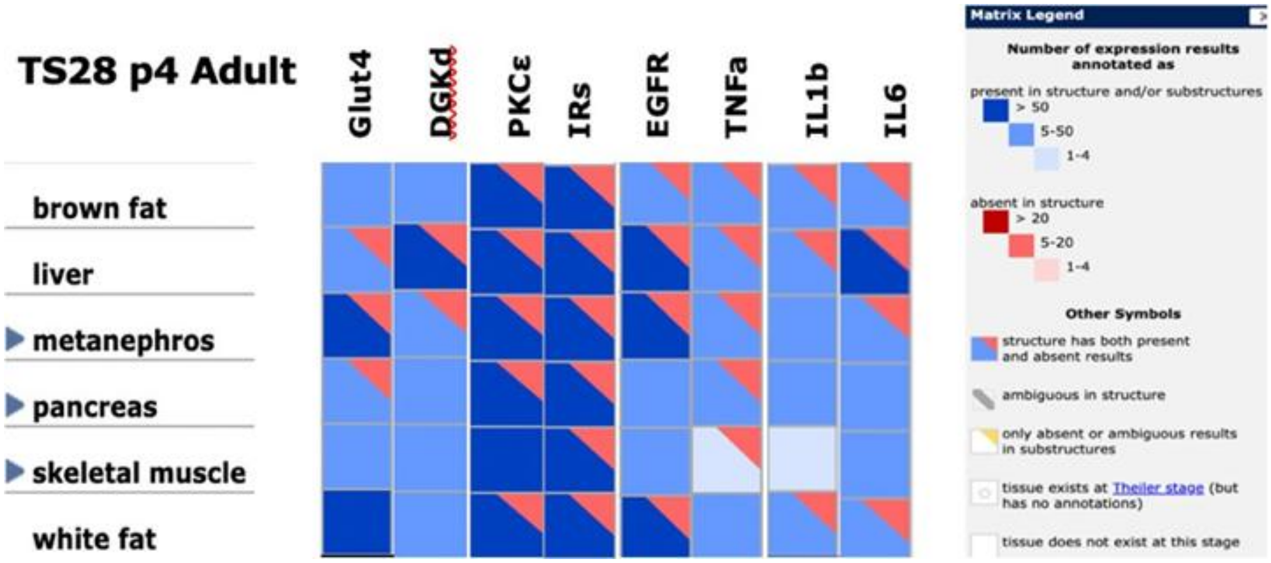

Supplementary Figure 1. Summary of the studied gene expression in the different studied tissues. The diagram was built based on the data from the Mouse Genome Database (MGD) and the Gene Expression Database (GXD). https://www.informatics.jax.org.

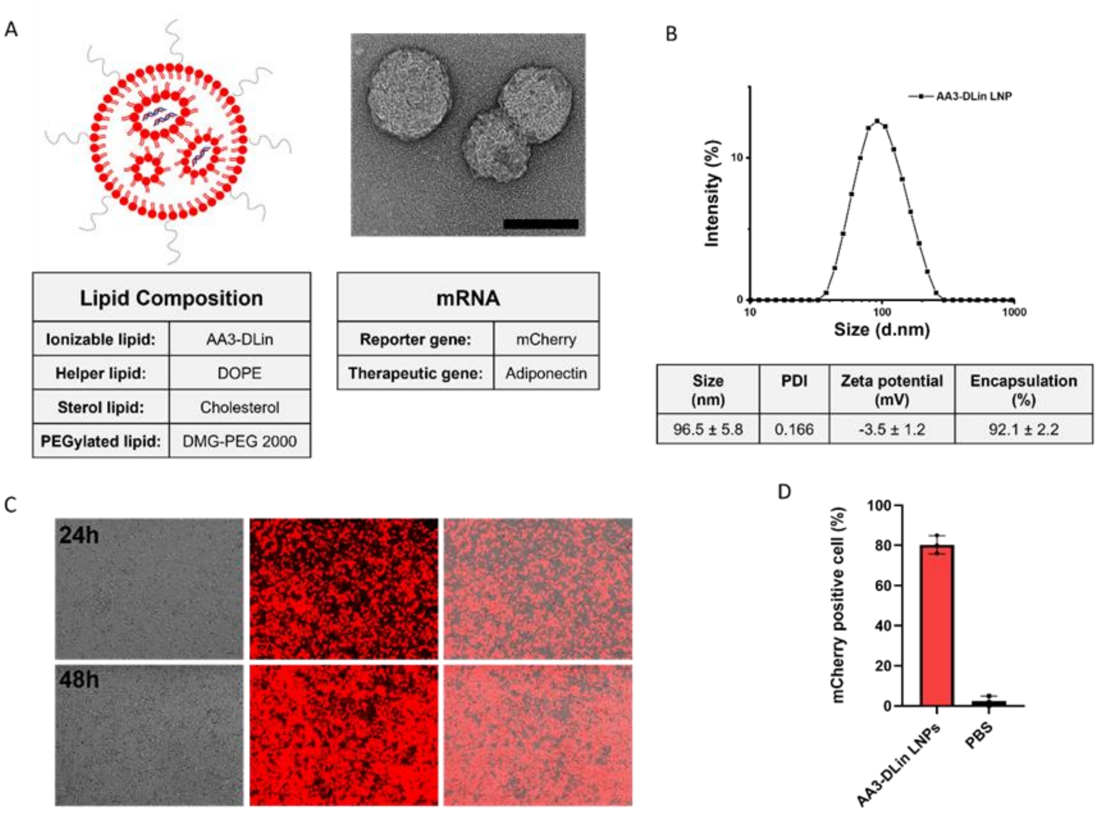

Supplementary Figure 2. Physicochemical properties, transfection evaluations, and intracellular uptake of the nanoparticles. A: A schematic to present the composition of AA3-DLin LNPs used in this study for mRNA delivery and a representative TEM image of LNPs (Scale bar: 100 nm). B: The physicochemical characterization of APN mRNA- LNPs: including size, polydispersity index (PDI), zeta, and mRNA encapsulation efficacy. C: The cell transfection efficacy of mCherry mRNA-LNPs, the cell images were captured by

© 2024. El-Araby RE et al. Published online at http://www.aginganddisease.org/EN/10.14336/AD.2024.0162

fluorescence microscope at pre-set time points. D: The population summary of mCherry-positive cells, compared to PBS as an empty control group. All experiments were repeated in triplicate and the results were represented as means ± SD.

Supplementary Table 1. Primer sequences of the studied genes.

APN	Forward	5′- GCACTGGCAAGTTCTACTGCAA -3′
	Reverse	5′- GTAGGTGAAGAGAACGGCCTTGT -3′
Glut-4	Forward	5′- GTAACTTCATTGTCGGCATGG -3′
	Reverse	5′- AGCTGAGATCTGGTCAAACG -3′
PKCε	Forward	5′-GCAGCAATAGAGTTGGGTTAG-3′
	Reverse	Reverse 5′- CAGGTTGTTCCGGATGTCC-3′
DGK-d	Forward	5′- GTGGTGATCTCATCAGCC -3′
	Reverse	5′- TCTTCTCAGATTCAGAGAGG -3′
EGFR	Forward	5′- GGAGGAAAAGAAAGTCTGCC -3′,
	Reverse	5′- ATCG- CACAGCACCA ATCAGG -3′
IR	Forward	5′-GAGAGGATGTGAGACGACG-3′
	Reverse	5′-AAGGTGTTAGGCAAAGGCAG-3′
TNF-α	Forward	5′-TCTCATGCACCACCATCAAGGACT-3′
	Reverse	5′-ACCACTCTCCCTTTGCAGAACTCA-3′
IL-1b	Forward	5′-AAGGGCTGCTTCCAAACCTTTGAC-3′
	Reverse	5′-ATACTGCCTGCCTGAAGCTCTTGT-3′
IL-6	Forward	5′-ATCCAGTTGCCTTCTTGGGACTGA-3′
	Reverse	5′-TAAGCCTCCGACTTGTGAAGTGGT-3′
